# Anatomic Study of the Elbow Joint in a Bengal Tiger (*Panthera tigris tigris*) Using Magnetic Resonance Imaging and Gross Dissections

**DOI:** 10.3390/ani9121058

**Published:** 2019-12-01

**Authors:** Mario Encinoso, Jorge Orós, Gregorio Ramírez, José Raduan Jaber, Alejandro Artiles, Alberto Arencibia

**Affiliations:** 1 Hospital Veterinario Los Tarahales, Recta de Los Tarahales 15, 35013 Las Palmas de Gran Canaria, Spain; mencinoso@gmail.com (M.E.); movilidad@hvtarahales.es (A.A.); 2 Departamento de Morfología, Facultad de Veterinaria, Universidad de Las Palmas de Gran Canaria, Trasmontaña, Arucas, 35416 Las Palmas, Spain; jorge.oros@ulpgc.es (J.O.); joseraduan.jaber@ulpgc.es (J.R.J.); 3 Departamento de Anatomía y Anatomía Patológica, Universidad de Murcia, 30100 Murcia, Spain; grzar@um.es

**Keywords:** magnetic resonance imaging, anatomy, elbow joint, Bengal tiger (*Panthera tigris tigris*)

## Abstract

**Simple Summary:**

We present a report describing the normal elbow joint anatomy in a Bengal tiger (*Panthera tigris tigris*) using magnetic resonance imaging (MRI) and gross dissections of this region. Anatomical findings detected using MRI of the different bony and soft tissues were evaluated according to the characteristics of signal intensity and compared with the corresponding gross anatomical dissections. The main anatomical structures were labelled and identified. This study provides a valuable resource for veterinarians, biologists, and researchers involved in Bengal tiger (*Panthera tigris tigris*) conservation.

**Abstract:**

The objective of our research was to describe the normal appearance of the bony and soft tissue structures of the elbow joint in a cadaver of a male mature Bengal tiger (*Panthera tigris tigris*) scanned via MRI. Using a 0.2 Tesla magnet, Spin-echo (SE) T1-weighting, and Gradient-echo short tau inversion recovery (GE-STIR), T2-weighting pulse sequences were selected to generate sagittal, transverse, and dorsal planes. In addition, gross dissections of the forelimb and its elbow joint were made. On anatomic dissections, all bony, articular, and muscular structures could be identified. The MRI images allowed us to observe the bony and many soft tissues of the tiger elbow joint. The SE T1-weighted MR images provided good anatomic detail of this joint, whereas the GE-STIR T2-weighted MR pulse sequence was best for synovial cavities. Detailed information is provided that may be used as initial anatomic reference for interpretation of MR images of the Bengal tiger (*Panthera tigris tigris*) elbow joint and in the diagnosis of disorders of this region.

## 1. Introduction

Magnetic resonance imaging (MRI) is commonly used, especially to assess the musculoskeletal system in humans and animals, due to its good image resolution, superior soft tissue contrast among different anatomical structures, and use of a magnetic field rather than ionizing radiation, compared with other imaging diagnostic technique [1,2,3]. In veterinary medicine, MRI anatomical studies have been performed in the elbow region of equines [4] and canines [5,6,7]. Several reports have provided descriptions of clinical findings regarding the elbow joint using MRI [8,9,10,11,12,13,14,15].

Since 2010, the Bengal tiger has been included on the Red List of Endangered Species [16]. In zoos and wildlife rehabilitation centers, veterinarians, researchers and specialized technicians are involved in large nondomestic cat medicine, as well as the welfare and conservation of this species. In tigers, anatomical and clinical studies via MRI are limited. To our knowledge, MRI has been only used to describe the normal anatomic features of the stifle and tarsus joints [17,18], as well as brain characteristics [19]. Also, previous reports describing several neurological disorders have been published on this species [20,21]. However, no published studies were found describing the Bengal tiger elbow joint using MRI and gross anatomical dissections.

The elbow joint is a synovial joint and is important due to its location of anatomical structures such as the articular surfaces of the bones (humerus, radius, and ulna), as well as the articular cavities, ligaments, muscles, and tendons that contribute to the stability of this region. The elbow is susceptible to congenital, developmental, and traumatic musculoskeletal disorders. Therefore, an accurate interpretation and thorough understanding of regional anatomy of the MRI elbow images could be useful in the evaluation and diagnosis of different elbow joint disorders in felines [22,23,24,25,26,27,28].

The aim of this study was to describe the elbow joint findings in a Bengal tiger using MRI and gross anatomical dissections.

## 2. Materials and Methods

### 2.1. Animals

In this research, the cadaver of a captive female six-year-old Bengal tiger (105 kg) was referred from the Cocodrilos Park Zoo (Canary Islands, Spain) to the Veterinary Faculty of Las Palmas de Gran Canaria University. The tiger, which had no history of any disease related to articulation, was subjected to observation, and no external elbow lesions were observed. After, the animal was immediately frozen to mitigate post-mortem changes. Two days later, the tiger was defrosted to perform the MRI study. This research was authorized by the Conservation Nature Service (Seprona) of Gran Canaria at the Spanish Ministry of Interior (Protocol 2012).

### 2.2. Magnetic Resonance Imaging

With the objective of carrying out the MRI, a 0.2-Tesla magnet (Vet-MR Esaote, Genoa, Italy) was employed with the tiger placed in right lateral recumbence. A standard MRI protocol was used to generate spin-echo (SE) T1-weighted and Gradient-Echo short tau inversion recovery (GE-STIR) T2-weighted images in the sagittal, transverse, and dorsal anatomical planes. The sagittal plane was aligned parallel to the olecranon tuberosity and planned using the transverse and dorsal plane. The transverse plane was perpendicular to the body of humerus and ulna and planned on the sagittal and dorsal planes. The dorsal plane was aligned parallel to the head of radius and planned using the sagittal and transverse planes.

SE T1-weighted sagittal images were acquired with the following settings: Echo time (TE) = 26 ms, repetition time (TR) = 700 ms, an acquisition matrix of 320 × 187, and 4-mm slice thickness with 4.4-mm spacing between slices. For GE-STIR T2-weighted sagittal images, the TE was 25 ms, TR was 1680 ms, acquisition matrix was 256 × 172, and 4-mm slice thickness with 4.4-mm interslice spacing was used. For SE T1-weighted dorsal images, the TE was 80 ms, TR was 3000 ms, acquisition matrix was 256 × 172, and 4.5-mm slice thickness with 4.9-mm interslice spacing was used. For GE-STIR T2-weighted dorsal images, the TE was 25 ms, TR was 1680 ms, acquisition matrix was 256 × 172, and 4.5-mm slice thickness with 4.9-mm interslice spacing was used. For SE T1-weighted transverse images, the TE was 26 ms, TR was 500 ms, acquisition matrix was 256 × 172, and 5-mm slice thickness with 5.5-mm interslice spacing. For GE-STIR T2-weighted transverse images, the TE was 25 ms, TR was 1680 ms, acquisition matrix was 256 × 172, and 5-mm slice thickness with 5.5-mm interslice spacing. In our study, a medical imaging software tool (OsiriX MD, http://www.osirix-viewer.com, Geneva, Switzerland) was used to evaluate the Dicom images obtained.

### 2.3. Anatomical Evaluation

After the performance of the imaging procedure, lateral and medial gross anatomical dissections of the right forelimb were performed in order to facilitate the identification and comparison of brachial and antebrachial muscles and tendons. Also, dissections of the articulation were made to facilitate the identification of the ligaments and bones. These images were compared to the corresponding MRI images presented in this study. Also, we also resorted to the Bengal elbow bones and to veterinary anatomy literature [29,30]. We labelled the identified elbow joint structures according to the anatomical nomenclature [31,32].

## 3. Results

### 3.1. Gross Anatomical Dissections

**Figure 1:**
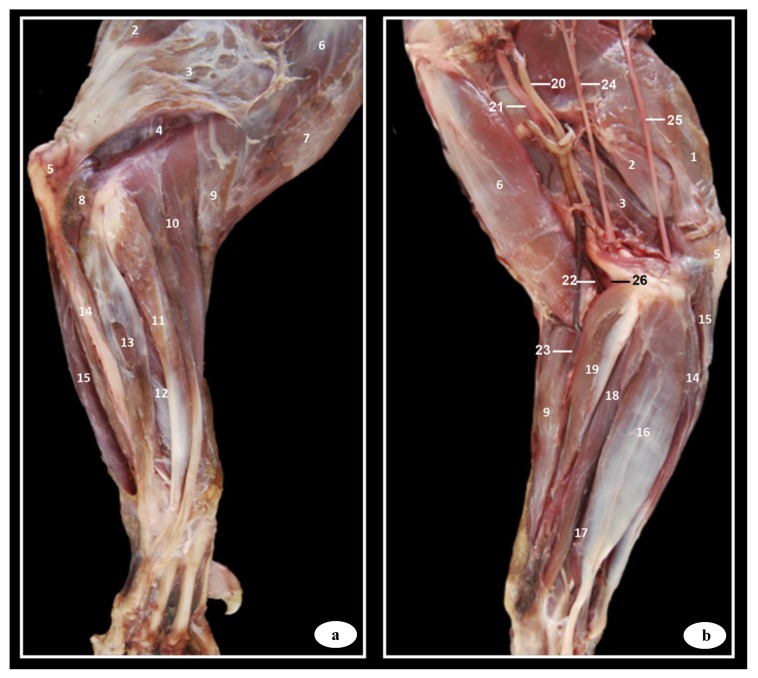
Dissection images of the Bengal tiger forelimb. (**a**) Lateral aspect and (**b**) medial aspect. 1. Tensor fasciae antebrachii muscle; 2. Triceps brachii muscle (long head); 3. Triceps brachii muscle (lateral head); 4. Triceps brachii muscle (accessory head); 5. Olecranon tuberosity; 6. Brachialis muscle; 7. Biceps brachii muscle; 8. Anconeus muscle; 9. Extensor carpi radialis muscle; 10. Extensor digitorum communis muscle; 11. Extensor digitorum lateralis muscle; 12. Abductor digiti I longus muscle; 13. Extensor carpi ulnaris muscle; 14. Flexor carpi ulnaris muscle (humeral head); 15. Flexor carpi ulnaris muscle (cubital head); 16. Flexor digitorum superficialis muscle; 17. Flexor digitorum profundus muscle; 18. Flexor carpi radialis muscle; 19. Pronator teres muscle; 20. Brachial vein; 21. Brachial artery; 22. Median artery; 23. Radial artery; 24. Radial nerve; 25. Cubital nerve; 26. Median nerve.

**Figure 2:**
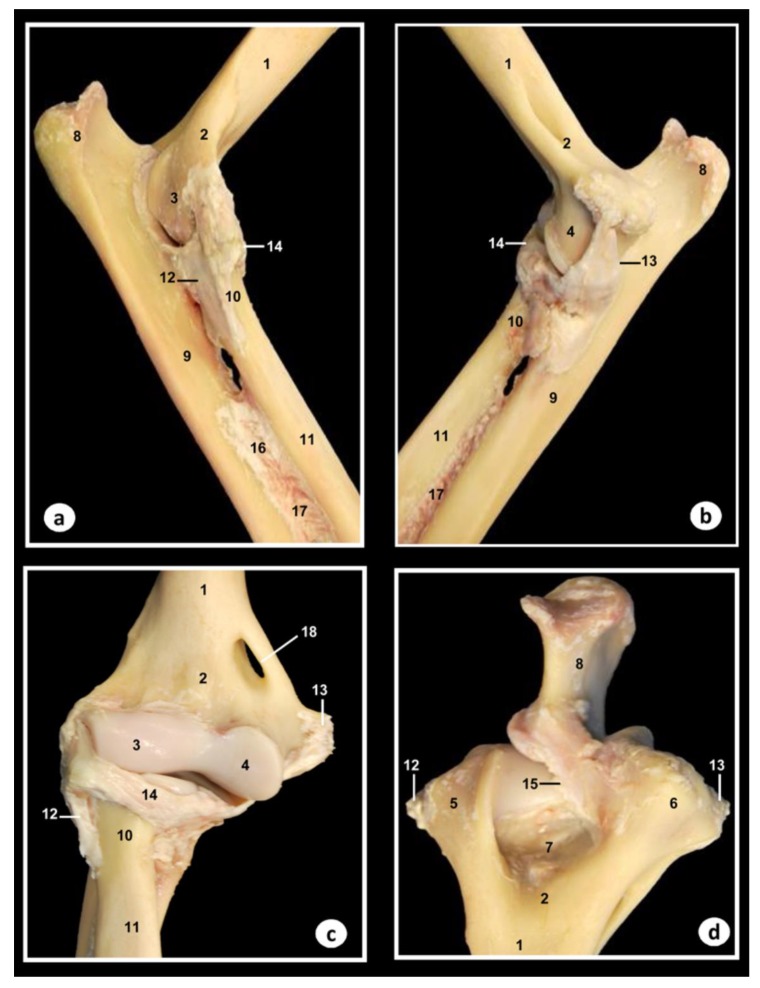
Dissection images of the Bengal tiger right elbow joint. (**a**) Lateral aspect, (**b**) medial aspect, (**c**) cranial aspect, and (**d**) proximal aspect. 1. Humerus (body); 2. Humerus (metaphysis); 3. Humerus (lateral condyle); 4. Humerus (medial condyle); 5. Humerus (lateral epicondyle); 6. Humerus (medial epicondyle); 7. Olecranon fossa; 8. Olecranon tuberosity; 9. Ulna (body); 10. Radius (head); 11. Radius (body); 12. Lateral collateral ligament; 13. Medial collateral ligament; 14. Radial annular ligament; 15. Olecranon ligament; 16. Interosseoum antebrachii ligament; 17. Membrana interossea antebrachia; 18. Supracondylar foramen.

Anatomical dissections images of the right forelimb and its corresponding elbow joint from different aspect are presented (Figure 1 and Figure 2). In Figure 1, the main muscles and tendons that stabilize this joint could be identified. Thus, the triceps brachii muscle and the muscular complex composed of the longum, lateral, medial, and accessory head were visible. The longum head originates from the caudal border of scapula, the lateral head originate lateral to the neck of humerus, the medial head originate from the humerus caudal to tuberositas teres major, and the accessory head originates to the neck of humerus. The longum, lateral, medial, and accessory head were identified with their tendons inserted into the olecranon tuberosity (Figure 1a,b). The anconeus muscle, extending from the humeral epicondyles and lateral to the olecranon fossa and terminating on the olecranon, was visible in Figure 1a. The extensor carpi radialis muscle was identified to extend from the lateral supracondylar crest of the humerus to the base of the II and III metacarpal bones (Figure 1a). The extensor digitorum communis muscle arose from the lateral epicondyle of humerus and the tendon split to terminate on the distal phalanx of second to fifth digits was also visible (Figure 1a). The extensor digitorum lateralis muscle was seen in Figure 1a from the lateral collateral ligament and radius head, and its tendons terminated on the III, IV, and V digits. The extensor carpi ulnaris muscle was visible from the lateral epicondyle of humerus, and it terminated on basis of the fifth metacarpal bone, and in the accessory and carpocubital bones (Figure 1a). The abductor digiti I longus muscle arose from the radius and ulna obliquely, which terminated on the basis of 1th metacarpal bone, was also identified in the Figure 1a.

In Figure 1b, the tensor fasciae antebrachii muscle was visible aponeurotic from the tendon of insertion of the latisimus dorsi muscle and terminated on the medial collateral ligament. The biceps brachii muscle, extending from the supraglenoidal tubercle and terminating on the radial tuberosity and medial collateral ligament, was seen in Figure 1a,b. The brachialis muscle was identified to extend from the caudal surface of the humerus to the radial tuberosity (Figure 1a). The flexor carpi radialis muscle was observed from the medial condyle of humerus to the second and third metacarpal bones (Figure 1b). The flexor digitorum superficialis muscle was visible from the medial epicondyle of humerus and terminated divided in four tendons on the middle phalanx of the second to fifth digits (Figure 1b). The flexor digitorum profundus muscle is composed of three heads with combined tendon, which divides to terminate on flexor surface of each distal phalanx (Figure 1b). The flexor carpi ulnaris muscle, composed by the humeral and ulnar heads, was visible in the Figure 1b. The humeral head was identified to extend from medial epicondyle of humerus to the carpal accessory bone, whereas the ulnar head was seen from medial side of olecranon to the carpal accessory bone (Figure 1b). The pronator teres muscle, extending from the medial epicondyle of humerus and terminating on the medial surface of radius, was also identified in the Figure 1b. Also, the brachial vein and artery, the median artery and nerve, the radial artery and nerve, and the cubital nerve were well-defined in the Figure 1b.

In Figure 2, corresponding to the gross dissections of the right elbow joint, several bones, ligaments, and membrane could be identified. Thus, the humerus bone (including the body, metaphysis, condyles, epicondyles, and the olecranon fossa), the radius bone (with their head, metaphysis, radial tuberosity, and articular circumference), and the ulna bone (olecranon tuberosity, anconeus process, lateral, and medial coronoid processes and body) were observed. Also, the supracondylar foramen is clearly visible in Figure 2c. Articular structures of elbow joint between humerus, radius, and ulna bones, such as the articular cavities, the lateral collateral cubital ligament that connects lateral epycondyle of humerus to radius, and the medial collateral cubital ligament that connects medial epycondyle of humerus to radius, were identified in Figure 2. Also, several structures of the proximal radiocubital joint were observed. Thus, the radial annular ligament that encircles the head of radius is attached to the edges of the radial incisure of the ulna was visible in Figure 2a–c, whereas the olecranon ligament from the medial border of olecranon fossa to olecranon was seen only in the proximal aspect (Figure 2d). In addition, the membrane interossea antebrachii that connects radius and ulna bones, and the interosseum antebrachii ligament that connects radius and ulna in the proximal half of spatium interosseum, were also visible in Figure 2a,b.

### 3.2. Magnetic Resonance Imaging

**Figure 3:**
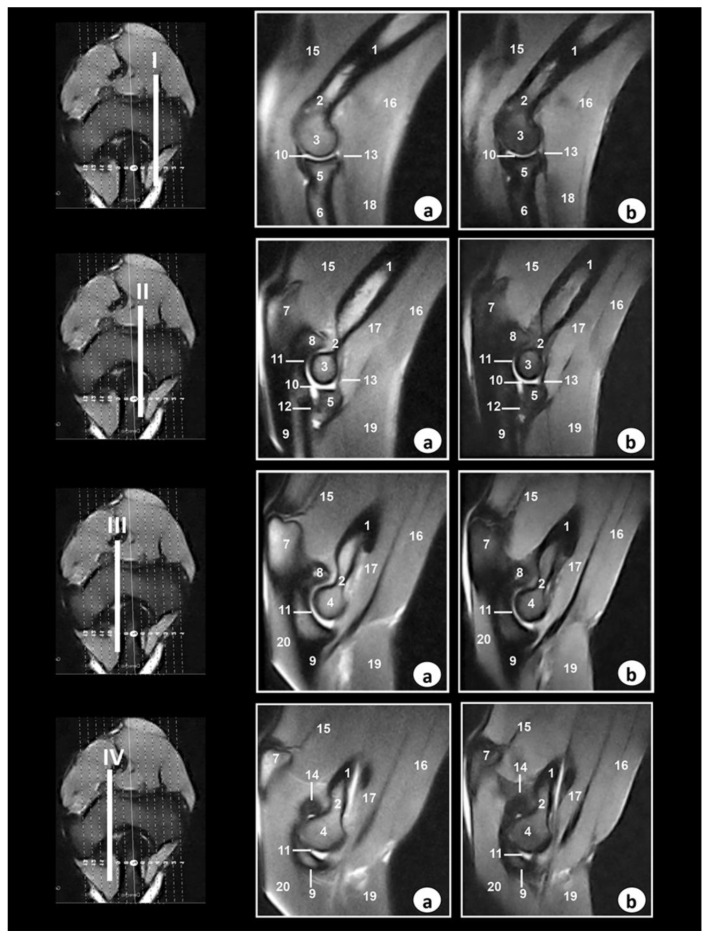
Sagittal MR images of the right elbow joint at the level of the lateral epicondyle of humerus (level I), lateral part of olecranon fossa (level II), medial part of olecranon fossa (level III), and medial epicondyle of humerus (level IV). The lines depict the level of section. The images are oriented so that the left is caudal and the right is cranial. (**a**) SE T1-weighted MR images and (**b**) GE-STIR T2-weighted images. 1. Humerus (body); 2. Humerus (metaphysis); 3. Humerus (lateral epicondyle); 4. Humerus (medial epicondyle); 5. Radius (head); 6. radius (body); 7. Ulna (olecranon tuberosity); 8. Ulna (anconeus process); 9. Ulna (body); 10. Humeroradial joint (articular cavity); 11. Humeroulnar joint (articular cavity); 12. Proximal radioulnar joint (articular cavity); 13. Radial annular ligament; 14. Olecranon ligament; 15. Triceps brachii muscle; 16. Biceps brachii muscle; 17. Brachialis muscle; 18. Extensor digitorum communis; 19. Extensor carpi radialis muscle; 20. Flexor carpi ulnaris muscle.

**Figure 4:**
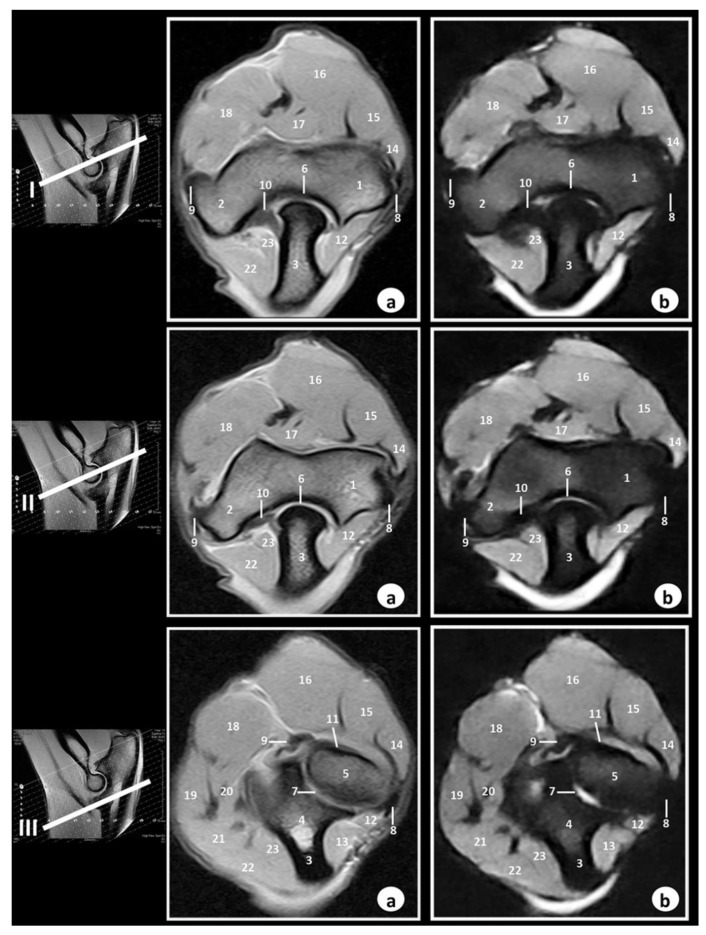
Transverse MR images of the right elbow joint at the level of olecranon tuberosity (level I), epicondyles of humerus (level II), and proximal radioulnar joint (level III). The lines depict the level of section. The images are oriented so that the left is medial, and the right is lateral. (**a**) SE T1-weighted MR images and (**b**) GE-STIR T2-weighted images. 1. Humerus (lateral epicondyle); 2. Humerus (medial epicondyle); 3. Ulna (olecranon tuberosity); 4. Ulna (body); 5. Radius head; 6. Humeroulnar joint (articular cavity); 7. Proximal radioulnar joint (articular cavity); 8. Lateral collateral ligament; 9. Medial collateral ligament; 10. Olecranon ligament; 11. Radial annular ligament; 12. Anconeus muscle; 13. Extensor carpi ulnaris muscle; 14. Extensor digitorum lateralis muscle; 15. Extensor digitorum communis muscle; 16. Extensor carpi radialis muscle; 17. Brachialis muscle; 18. Biceps brachii muscle; 19. Flexor digitorum superficialis muscle; 20. Flexor carpi radialis muscle; 21. Flexor digitorum profundus muscle; 22. fFexor carpi ulnaris muscle (humeral head); 23. Flexor carpi ulnaris muscle (ulnar head).

**Figure 5:**
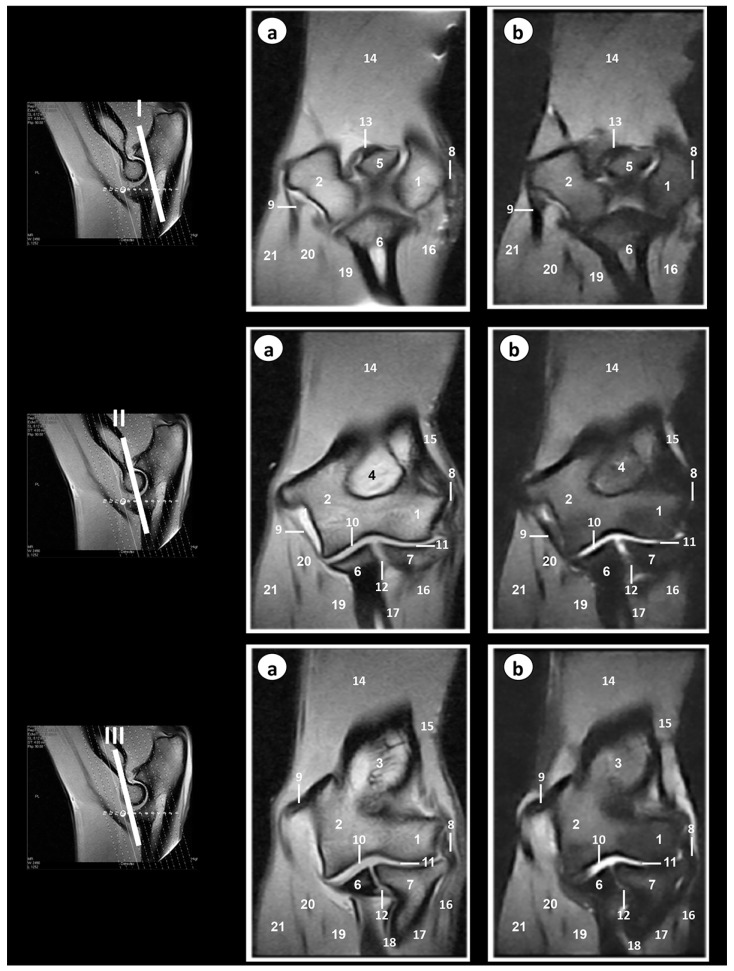
Dorsal MR images of the right elbow joint at the level of anconeus processus of ulna (level I), olecranon fossa (level II), and head of radius (level III). The lines depict the level of section. The images are oriented so that the right side of the image is lateral and the top is proximal. (**a**) SE T1-weighted MR images and (**b**) GE-STIR T2-weighted images. 1. Humerus (lateral epicondyle); 2. Humerus (medial epicondyle); 3. Humerus (metaphysis); 4. Olecranon fossa; 5. Anconeus process; 6. Ulna (body); 7. Radius (head); 8. Lateral collateral ligament; 9. Medial collateral ligament; 10. Humeroulnar joint (articular cavity); 11. Humerorradial joint (articular cavity); 12. Proximal radioulnar joint (articular cavity); 13. Olecranon ligament; 14. Triceps brachii muscle; 15. Anconeus muscle; 16. Extensor digitorum lateralis muscle; 17. Extensor digitorum communis muscle; 18. Extensor carpi ulnaris muscle; 19. Flexor digitorum profundus muscle; 20. Flexor carpi ulnaris muscle; 21. Flexor digitorum superficialis muscle.

Selected MR images are presented in Figure 3, Figure 4 and Figure 5. In Figure 3, four sagittal MR images are shown in a lateromedial direction from the lateral epicondyle of humerus (level I) to medial epicondyle of humerus (level IV).

Figure 4 shows three transverse MR images presented in a proximodistal direction from the olecranon tuberosity (level I) to the proximal radioulnar joint (level III).

In Figure 5, three dorsal MR images are presented in a caudocranial direction from the anconeus process of humerus (level I) to the head of radius (level III).

On the MR images, anatomic details of the Bengal tiger elbow joint were evaluated according to the characteristics of signal intensity of the different bony and soft tissues (Table 1).

In the SE T1-weighted sequence, the cortical and subchondral bone of the humerus, radius, and ulna appeared with very low signal intensity compared with the high signal of the bone marrow. The articular cartilage was visualized with intermediate signal intensity. By contrast, in the GE-STIR T2-weighted MR images, the bone marrow was seen with intermediate signal intensity and could be observed in the area of the negligible signal corresponding to the cortical and subchondral bones. The articular cartilage was identified by the intermediate signal characteristics on both the SE T1-weighted and GE-STIR T2-weighted images (Figure 3, Figure 4 and Figure 5). The lateral and medial collateral ligaments of the elbow joint were readily seen on the transverse (Figure 4) and dorsal (Figure 5) planes. These ligaments were visible with low signal intensity in SE-T1-weighted images and with very low signal in the GE-STIR T2-weighted pulse sequence as linear bands similar in intensity to the cortical bone. On the other hand, articular structures of radiocubital proximal joint were also visible in the images. Thus, the radial annular ligament was observed in Figure 4 and Figure 5, whereas the olecranon ligament was more clearly identified in the sagittal (Figure 3) and transverse (Figure 4) images. Besides, the membrana interossea antebrachii and interosseoum antebrachii ligament were seen especially in sagittal MR images (Figure 3). This ligament and membrane had low signal intensity in both sequences. Finally, synovia could be seen in the articular cavities with intermediate signal intensity on the T1-weighted MR images. By contrast, in the GE-STIR T2-weighted images, synovial fluid appeared with high signal intensity. Several main muscles, such as the brachialis, the antebrachial fascia tensor, biceps brachii, the triceps brachii, anconeus, extensor carpi radialis, extensor digitorum communis, extensor digitorum lateralis, abductor digiti I longus, extensor carpi ulnaris, flexor carpi ulnaris, flexor digitorum superficialis, flexor digitorum profundus, flexor carpi radialis, and pronator teres, were well-identified in Figure 3, Figure 4 and Figure 5. These muscles were defined with intermediate signal intensity in both sequences. By contrast, its tendons appeared with dark grey to black signal intensities in the SE T1-weighted images and with dark grey signal intensity in the GE STIR T2-weighted images. The muscles and tendons were easily seen, especially in the sagittal (Figure 3) and transverse (Figure 4) images, compared with the dorsal plane (Figure 5).

## 4. Discussion

In humans, MRI has become the modality of choice for morphological assessment and the diagnosis and management of diseases of the elbow joint [33]. Advances in equipment and MRI pulse sequences have allowed for superior visualization of this joint and its adjacent structures. Numerous MRI pulse sequences have been applied to musculoskeletal system. Traditionally, spin-echo sequences have been used for elbow diagnostic imaging. However, these techniques have been supplanted by the newer fast spin-echo and gradient-echo pulse sequences, which provide anatomical and functional information with greater ability to distinguish between bony and soft tissue structures [33,34].

In veterinary practice, the exploration of anatomic structures within the elbow joint and clinical assessment of soft tissues is laborious because of the anatomic complexity. Traditionally, radiography [35] and ultrasonography [36] have been used to obtain images of the bony and the main soft-tissue structures of this region. Nevertheless, computed tomography has become the preferred imaging technique for the evaluation of the osseous structures [37] and MRI has progressively gained credit for their ability to assess the ligaments, musculotendinous, cartilaginous surfaces, and osseous structures of this anatomical region [4,5,6,7].

In recent years, the contributions of the veterinarians working with captive and free-ranging animals to prevent and/or treat diseases that threaten species’ survival in wildlife conservation have increased [38]. In the Bengal tiger (*Panthera tigris tigris*), as well as in other mammals, the elbow conforms an anatomical region composed by bones, ligaments, muscles, and tendons that support this joint. Thus, the physical examinations and clinical assessments of these structures are very difficult due to its complexity. Our MRI study facilitated the identification of the main elbow anatomical structures. Also, an accurate anatomical interpretation of MRI images would be useful for assessment of elbow joint tissues such as bones, fluids, ligaments, and muscular structures, and could be used in the diagnosis of disorders of this joint.

The interpretation of elbow joint MRI studies requires an understanding of the MRI unit, basic pulse sequences, and standard imaging planes [33]. With regard to the MRI protocol used in the present study, an MRI can be used as an initial valid reference for assessment of the Bengal tiger (*Panthera tigris tigris*) elbow. However, more clinical studies, which include more specimens to assess the adequate MRI protocols for elbow disorders, are necessary. Our research was obtained via low-field MRI magnet (0.2 T), which provided a correct visualization of the main anatomical structures of this joint. Anatomical characteristics of the elbow joint using low-field MRI have been reported in dogs [6], and previous studies have evaluated this anatomical region using high-field magnet in horses [4] and dogs [5,7]. Low-field (0.2–0.4 T) MRI equipment predominates in veterinary practice due to its reduced costs, better patient access, and greater safety compared to high-field MRI units [38]. However, in veterinary musculoskeletal imaging, using high-field equipment is recommended. High-field equipment improves the signal-to-noise ratio, resulting in increased image resolution and decreased exam time of bones, ligaments, muscles, and tendons [39,40].

Manipulation of the technical parameters permits a variety of MRI pulse sequences. The SE T1-weighted MR images shows fat as a bright signal intensity. By contrast, the GE-STIR-T2-weighted sequence is a fat-suppression technique and shows water as a bright signal intensity [34]. In our study, these same MRI pulse sequences were selected. SE-T1-weighted MR images were best to distinguish the best anatomic detail, whereas GE-STIR-T2-weighted MR images showed anatomical information for synovial cavities of the humeroradial, humeroulnar, and proximal radioulnar joints. Nevertheless, in both MRI sequences, the major difficulty was the definition of articular cartilage due to the presence of synovial fluid with similar MRI signal intensity. The elbow joints of dogs [5,6,7] and horses [4] have been studied using similar MRI pulse sequences.

In our research, the Bengal tiger (*Panthera tigris tigris*) elbow joint was imaged in three anatomical planes: Sagittal, transverse, and dorsal. In this regard, Baeumlin et al. [6] reported these same planes in dogs [6], whereas Snaps et al. [5] and Wucherer et al. [7] showed images of the elbow joint in dogs only in the sagittal and dorsal planes, respectively. On the other hand, Tnibar et al. [4] showed images of this region in the horses only in the transverse plane. In our study, the images allowed us to see the relationship between the cortical, subchondral bone, and bone marrow. Several ligaments were identified in the MRI planes. Thus, the lateral and medial collateral ligaments of the elbow joint showed the best views in the dorsal plane. These observations have also been reported in horses [4] and dogs [5,6,7]. On the other hand, the transverse and dorsal planes provided better definition of the olecranon ligament, whereas the radial annular ligament was observed especially in the sagittal and transverse planes. Also, in our research, the sagittal planes provided optimal views of the proximal radioulnar joint structures, such as the membrana interossea antebrachii and interosseoum antebrachii ligament. These articular structures have not described previously. Moreover, the brachii, extensor, and flexor muscles were best seen in the different planes. Thus, the biceps brachii, brachialis, and triceps brachii were best seen on the sagittal plane. The extensor muscles of the forelimb were seen in the transverse and dorsal planes, whereas the flexor muscles were visible especially in the sagittal and dorsal planes. However, the tendons of these muscles were not visible in all images because of their very short size. These observations were previously described in dogs [6].

The efficiency and effectiveness of conservation efforts are significantly enhanced by incorporating animal health considerations into the planning, implementation, and evaluation phases of all programs (captive and wild) involved in conserving wildlife [38]. Anatomical and clinical studies on captive felines are essential activities for tiger conservation around the world. The identification of the bony, articular cavities, ligaments, muscles, and tendons presented in this study was facilitated by the use of gross anatomical dissections of the right forelimb and elbow joint. The gross anatomical dissections provided a good location of the main anatomical structures, and they are a helpful tool for the identification of MRI features. Obtaining MRIs of the Bengal tiger is severely hindered by high cost and limited availability. Nevertheless, the small risk degree which its application entails might allow us to justify its use in these endangered species. With developing technology in zoos and wildlife rehabilitation centres, MRI is a promising, noninvasive, and accurate method for tiger imaging [17,18,19,20,21]. Our research provides the first anatomical description of the elbow in a Bengal tiger (*Panthera tigris tigris*) via low-field MRI. In the future, it is important to carry out new studies and to establish new MRI protocols to ensure a better assessment and diagnosis of disorders of this joint using low-field and high-field MRI units.

## 5. Conclusions

In conclusion, imaging findings of this research indicate that MRI provides an accurate depiction of the anatomical structures of the elbow joint. These images should be useful for veterinarians, biologists, researchers, and technicians involved in Bengal tiger (*Panthera tigris tigris*) medicine, welfare, and conservation.

## Figures and Tables

**Table 1:** Tissue signal intensity characteristics for MRI of the Bengal tiger (*Panthera tigris tigris*) elbow joint.

TISSUE	SE-T1 Weighted	GE-STIR-T2 Weighted
**Cortical and subchondral bone**	Very low	Very low
**Bone marrow**	High	Intermediate
**Fat**	High	Intermediate
**Synovial fluid**	Low	High
**Articular cartilage**	Intermediate	Intermediate
**Ligament**	Low	Very low
**Muscle**	Intermediate	Intermediate
**Tendon**	Low	Very low
